# Precision Medicine and Adverse Drug Reactions Related to Cardiovascular Drugs †

**DOI:** 10.3390/diseases9030055

**Published:** 2021-08-12

**Authors:** James D. Noyes, Ify R. Mordi, Alexander S. Doney, Rahman Jamal, Chim C. Lang

**Affiliations:** 1 Division of Molecular and Clinical Medicine, School of Medicine, University of Dundee, Dundee DD1 9SY, UK; james.noyes@nhs.scot (J.D.N.); i.mordi@dundee.ac.uk (I.R.M.); 2 Division of Population Health and Genomics, School of Medicine, University of Dundee, Dundee DD1 9SY, UK; a.doney@dundee.ac.uk; 3 UKM Medical Molecular Biology Institute, Universiti Kebangsaan Malaysia Medical Centre, Kuala Lumpur 56000, Malaysia; rahmanj@ppukm.ukm.edu.my

**Keywords:** precision medicine, adverse drug reaction, cardiology

## Abstract

Cardiovascular disease remains the leading global cause of death. Early intervention, with lifestyle advice alongside appropriate medical therapies, is fundamental to reduce patient mortality among high-risk individuals. For those who live with the daily challenges of cardiovascular disease, pharmacological management aims to relieve symptoms and prevent disease progression. Despite best efforts, prescription drugs are not without their adverse effects, which can cause significant patient morbidity and consequential economic burden for healthcare systems. Patients with cardiovascular diseases are often among the most vulnerable to adverse drug reactions due to multiple co-morbidities and advanced age. Examining a patient’s genome to assess for variants that may alter drug efficacy and susceptibility to adverse reactions underpins pharmacogenomics. This strategy is increasingly being implemented in clinical cardiology to tailor patient therapies. The identification of specific variants associated with adverse drug effects aims to predict those at greatest risk of harm, allowing alternative therapies to be given. This review will explore current guidance available for pharmacogenomic-based prescribing as well as exploring the potential implementation of genetic risk scores to tailor treatment. The benefits of large databases and electronic health records will be discussed to help facilitate the integration of pharmacogenomics into primary care, the heartland of prescribing.

## 1. Introduction

The term ‘patient-centred care’ resonates throughout healthcare settings, providing a constant reminder to tailor treatments to individual patients. Despite clinical similarities, retaining the knowledge that patients may react differently to the same intervention underpins the importance of precision medicine. This is true for both pharmacological and non-pharmacological treatments. Over the last twenty years, our knowledge of why certain therapies work well on some, but not others, has improved. Recognised initially through anecdotal phenotypic variation, advances in genomics have provided the mechanistic insight to uncover drug-gene interactions. Pharmacogenomics is an exciting and fast-moving field that has already broken through into drug regulatory approval bodies. However, much work remains in order to fully integrate these scientific advances at the bedside. This review aims to address the importance of the clinical implementation of pharmacogenomics to reduce patient harm. Additionally, the strategies available to help incorporate pharmacogenomic-based prescribing into clinical practice will be discussed.

Cardiovascular disease is the leading cause of death in the United Kingdom, and multiple pharmacological therapies are prescribed to vast quantities of patients for both primary and secondary prevention of disease. For this reason, cardiovascular therapies will be the focus of this review. Despite numerous trials showing the population benefits of lowering cardiovascular disease risk with pharmacological treatments, it is important to remember that many patients experience significant iatrogenic harm. A systematic review identified that between 4.6% and 12.1% of hospitalisations were attributed to medicine related problems. Interestingly, they also found that cardiovascular and diabetes medicines were most often responsible [1]. Further studies have shown that the median bed stay in a hospital from an adverse drug reaction is 8 days, resulting in a predicted annual cost to the NHS of a staggering £466 million [2]. As our main population demographic continues to age, this figure is likely to increase significantly over the coming years.

## 2. Pharmacological Principles

**Figure 1:**
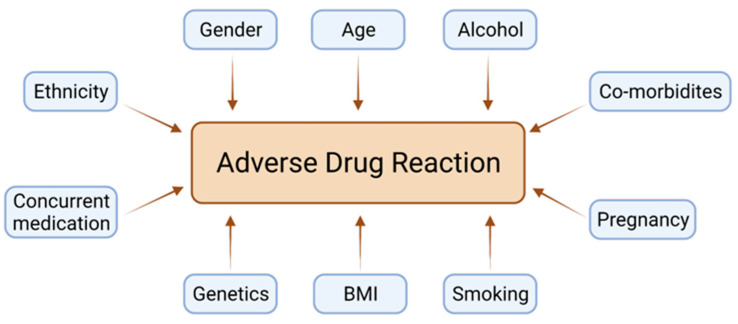
Contributing factors to an adverse drug reaction.

Given the scale of the problem, it is important to fully appreciate the underlying mechanisms behind an adverse drug reaction. There are many complex factors and interactions which ultimately contribute to this (Figure 1). However, a basic understanding of pharmacological principles helps to clarify the role that genetic variation plays. Pharmacokinetics describes the actions that the body carries out after exposure to a drug. There are four main actions: absorption, distribution, metabolism and excretion. These processes are dependable on enzymes and co-transporters, which are susceptible to interindividual variation based on differences in the protein-coding sections of genes. The clinical effects of such changes can be significant, resulting in harm from either under or over exposure to the drug’s therapeutic action.

Differences in genetic code can also influence how a drug acts in the body, which underpins the drug’s pharmacodynamic properties. The receptor on which a drug acts on is comprised of proteins that are subject to structural changes based on genetic variation. This can markedly alter the clinical response from the drug as described before. These examples depict the mechanisms underlying an ‘on-target’ adverse drug reaction. Nevertheless, despite advances in design and manufacturing, drugs are not entirely specific to their intended target receptor. They often act on numerous other receptors throughout the body with varying clinical significance. Alterations in ‘off-target’ receptors, caused by genetic variants, can result in unusual adverse effects which are difficult to predict. It is also true that increased drug concentration, caused by a genetic variation to the pharmacokinetic processing, can induce further ‘off-target’ adverse effects, an example of which includes myotoxicity in simvastatin use [3].

## 3. Clinical Guidance Available for Pharmacogenomic Based Prescribing

In the United Kingdom (UK), best practice guidelines are produced by the National Institute of Health and Care Excellence (NICE, London, UK) and in Scotland, by the Scottish Intercollegiate Guidelines Network (SIGN, Edinburgh, Scotland). Cardiology pharmacogenomic prescribing guidance has not filtered through to either at the time of writing. However, in the United States of America (USA), the Food and Drug Administration (FDA, Silver Spring, MD, USA) includes genetic information in its drug labelling for 15 cardiology drugs in addition to warfarin which is included under haematology medications [4]. Several groups have been instrumental in evaluating pharmacological and clinical evidence to produce high quality pharmacogenomic based guidance for prescribers. These include The Clinical Pharmacogenetics Implementation Consortium (CPIC, Stanford, CA, USA), The Royal Dutch Pharmacists Association—Pharmacogenetics Working Group (DPWG, Stanford, CA, USA) and The Canadian Pharmacogenomics Network for Drug Safety (CPNDS, Vancouver, BC, Canada). These recommendations have been subsequently annotated by PharmGKB, creating an easy-to-use resource that is free to access [5]. The cardiovascular therapies with the greatest pharmacogenomic evidence base are warfarin, clopidogrel and simvastatin. These drugs will be explored in greater detail below.

### 3.1. Warfarin

The vitamin K antagonist, warfarin, remains widely used in cardiology for managing a variety of conditions from atrial fibrillation to metallic heart valves. The anticoagulant effect arises from the inhibition of vitamin K dependent coagulation factors (II, VII, IX, X). Harm to the patient can occur from both under and over exposure to warfarin, causing an increased risk of either thromboembolic disease or bleeding, respectively. Pharmacogenomic studies have identified distinct genetic variants that are associated with both forms of adverse drug reaction. The CPIC provides further information and guidance regarding the variation of the following culprit genes: CYP2C9, VKORC1, and CYP4F2.

To appreciate the role that CYP2C9 gene variants contribute to warfarin’s adverse effects, it is first essential to note that warfarin is comprised of two stereoisomers: S-warfarin and R-warfarin. The CYP2C9 gene encodes the enzyme which metabolises S-warfarin, acting to reduce its concentration in the body. In total, 18 alleles of this gene have been found to reduce the function of the enzyme [6]. For affected patients, this results in higher levels of S-warfarin, the more potent of the isomers, increasing the risk of bleeding. There are two reduced function alleles, in particular, to be aware of: CYP2C9*2 and CYP2C9*3. These are the most common in European cohorts and have been shown to markedly reduce the metabolism of S-warfarin [7].

Variations in VKORC1 can also result in an increased bleeding risk to patients. The VKORC1 gene encodes the target receptor protein for warfarin, and alterations to this result in a pharmacodynamic ‘on-target’ adverse drug reaction. An example of this includes the common rs9923231 reduction-of-expression variant. Patients who have this variant require much lower doses of warfarin due to increased drug sensitivity [6,8].

Results from a large, double blinded, randomised control trial in patients with atrial fibrillation show the clinical benefit of using pharmacogenomics to guide therapy choice. Patients are most at risk of coming to harm from warfarin therapy in the early stages of treatment prior to establishing a safe dose based on the international normalised ratio (INR). The study showed that patients who were warfarin sensitive or highly sensitive, based on CYP2C9 and VKORC1 genetic variants, had a greater reduction in bleeding risk than normal responders when treated with edoxaban (a novel oral anticoagulant) compared with warfarin during the first 90 days of treatment. The study also showed that patients with CYP2C9 and VKORC1 genetic variants were more likely to experience early bleeding on warfarin compared to normal responders [9].

Conversely, the final genetic variant to discuss requires an increased dose of warfarin to counter its physiological effects. The CYP4F2 gene acts to reduce active vitamin K levels in the body. Alterations in this gene, including the CYP4F2*3 variant, disrupt this process leading to higher levels of vitamin K. Higher doses of warfarin are therefore needed to have a therapeutic effect, increasing the risk of thromboembolic disease in affected patients. An additional randomised control trial has been completed, incorporating all of the discussed genetic variants, to establish if prescribing safety of warfarin can be improved. The study was carried out on patients undergoing elective orthopaedic procedures requiring perioperative anticoagulation. They found that dosing warfarin based on genomic data reduced their risk of major bleeding, high INR, thromboembolic events and death compared to clinically guided dosing [10].

Although most clinicians are moving away from warfarin to alternative novel oral anticoagulation therapies, warfarin is still recommended in clinical practice guidelines for mechanical valve replacements and in conditions where there is no data for direct oral anticoagulants such as left ventricular thrombus. It is worth noting that many transferable lessons can be learned from the use of pharmacogenomics in warfarin prescribing. Additionally, warfarin studies have demonstrated that for certain patients, alternative medications should be considered to prevent adverse drug reactions.

### 3.2. Clopidogrel

Clopidogrel is an antiplatelet drug commonly prescribed in both cardiology and stroke medicine. However, its efficacy can be dramatically impaired by gene variants. The liver converts clopidogrel from its inactive prodrug form into active metabolites, which act to irreversibly block the purinergic P2RY_12_ receptor. This action prevents platelets from amassing to form platelet clumps for the duration of their 10-day life span [11]. The successful activation of clopidogrel in the liver requires the presence of a number of enzymes, including the cytochrome P450 (CYP)2C19 enzyme. A single loss of function mutation in the CYP2C19*2 (c.681G > A, rs4244285) gene has been associated with reduced drug effectiveness among European populations. Alterations in this gene result in a non-functioning hepatic enzyme, preventing the conversion of clopidogrel into its active metabolites. Around 25% of Caucasians possess this variant which increases their risk of an arterial thrombus when treated with clopidogrel [11,12].

Randomised control trials have been carried out to assess the potential benefits of genotyping patients prior to antiplatelet therapy. One such trial was carried out in a group of patients undergoing primary percutaneous coronary intervention with stent insertion. Patients were randomised to either the standard group in which they were treated with either ticagrelor or prasugrel; or the genotyped group in which those with loss of function mutation alleles also received ticagrelor or prasugrel and the others clopidogrel. The study found that those in the genotyped group had a lower incidence of bleeding and showed non-inferiority to standard treatment [13]. In the more recent TAILOR-PCI, which was an open labelled RCT comparing the genotype-guided choice of oral P2Y12 inhibitors to clopidogrel, as part of dual antiplatelet therapy after percutaneous coronary intervention, in people with CYP2C19 loss-of-function variants, there was also the suggestion of benefit. The primary outcome (composite of cardiovascular death, myocardial infarction, stroke, definite or probable stent thrombosis) occurred in 4.0% of the genotype-guided group and in 5.9% of the control group (hazard ratio, 0.66; *p* = 0.06) [14].

Acute cardiac events are most often treated with dual antiplatelet agents; the addition of aspirin provides a degree of protection from thrombotic complications. However, in the UK, the antiplatelet of choice for non-cardioembolic ischaemic stroke disease is clopidogrel monotherapy [15]. Patients who have an ischaemic stroke in the UK who carry the CYP2C19*2 loss of function allele are at particularly high risk of further events. A study carried out in Dundee explored this in more detail utilising observational data. Electronic medical records were used to identify patients hospitalised for an arterial thrombo-occlusive (ATO) event who then received clopidogrel prescriptions in the community. These patients were then followed up for 2 years. A primary endpoint of further ATO event or death was used, which occurred in 46% of the 651 participants. The study identified that those with the CYP2C19*2 loss of function allele had an increased risk of primary endpoint (hazard ratio (HR) 1.29). Further sub-analyses found this risk greatly increased (HR 2.23) among those diagnosed with an ischaemic stroke [12]. This study highlights, using real world data, that stroke patients with loss of function mutations of CYP2C19*2 are particularly susceptible to harm from reduced efficacy of clopidogrel treatment. Based on these data, there is a precision medicine care pathway for stroke patients in Dundee (https://www.registerforshare.org/whats-new/share-stroke-study-receives-100000-funding) (accessed on 1 May 2021).

### 3.3. Statins

One of the most widely cited examples of drug intolerance relates to statin therapy and muscle toxicity. A single nucleotide polymorphism (SNP) has been identified, which is associated with an increased systemic concentration of simvastatin, increasing the risk of muscle toxicity. The SNP is found in the SLCO1B1 gene, rs1419056T > C, and results in reduced activity of the OATP1B1 transporter in the liver. Reduced hepatic uptake of simvastatin results in larger systemic concentrations [16]. Interestingly, despite known associations between SLCO1B1 and other statins, such as atorvastatin, the link to muscle toxicity is much weaker [8].

One potential argument against incorporating pharmacogenomic based statin prescribing is that patients could be underdosed and come to significant harm as a result of this. The first randomised control trial addressing this issue has recently been reported. In a group of 408 patients, they found that there was no worsening of low-density lipoprotein cholesterol levels in the group whose physicians knew their SLCO1B1 results compared to the control group [17]. The findings do not advocate the widespread implementation of pharmacogenomic based statin prescribing; however, they can act to reassure those with concerns regarding poorer patient outcomes. All prescriptions in the study were in line with the CPIC guidelines, which recommend to either prescribe a lower dose of simvastatin or an alternative, such as pravastatin or rosuvastatin, in patients with an intermediate or low function SLCO1B1 phenotype [16].

## 4. Future Potential Targets for Pharmacogenomic Based Prescribing

Pharmacogenomics is a rapidly evolving field that looks ahead to the discoveries of tomorrow. The next section will focus on a selection of developing studies and aims to demonstrate the scope of pharmacogenomics and its novel application.

### 4.1. Angiotensin Converting Enzyme Inhibitors

Angiotensin-converting enzyme inhibitors (ACEi) are commonly prescribed by cardiologists for the management of hypertension and heart failure. Whilst ACEi related cough is well recognised by most healthcare professions, the severe complication of angioedema is not. Angioedema can present as a medical emergency with swelling of the face and mucosal membranes resulting in airway obstruction. It has an incidence of 0.1–0.7% and is not thought to be related to drug dose. Understanding the exact mechanism underlying this adverse drug event is still in progress; however, pharmacogenetics plays an important role.

Bradykinin levels have been found to be much higher in individuals suffering from ACEi angioedema compared to people on ACEi without angioedema. Bradykinin is usually broken down by ACE in the body; however, when inhibited by ACEi, alternative enzymes such as aminopeptidase P (APP) are relied upon to a greater extent. The XPNPEP2 gene codes for APP, and it has been shown that a variant of this gene was more prevalent in cases of ACEi angioedema compared to controls [18]. In addition to bradykinin, substance P has also been considered to play a contributory role in ACEi angioedema. This is a substrate of ACE and is associated with tissue swelling in mice. Substance P is broken down by the enzymes dipeptidyl peptidase 4 (DPP IV) and neutral endopeptidase (NEP). Studies have shown that the rs989692 variant in the MME gene, which codes for NEP, was found to be significantly associated with ACEi angioedema [19]. Further studies in the field have subsequently raised doubt about the causal effect of these gene variants, citing alternative factors such as gender and ethnicity, which appear to have a greater influence. Whilst these gene variations are likely to contribute to ACEi angioedema, they are not the dominant force driving the process [20]. Additional work is therefore required to underpin the exact mechanism responsible for this adverse drug reaction, yet pharmacogenomics has played a valuable contributory role to date.

### 4.2. Antibiotic Prescribing

The risk of adverse events from commonly prescribed cardiovascular medicines has been explored in detail above. However, a recent study has shown the benefits of using pharmacogenomics to establish those at increased risk of cardiovascular hospitalisation from commonly prescribed antibiotic therapy.

Trial data has shown that clarithromycin, a commonly prescribed macrolide antibiotic, is associated with an increased risk of cardiovascular mortality. Compared to placebo, 2 weeks of treatment with clarithromycin was associated with a 45% increase in relative risk of cardiovascular mortality [21]. Conversely, more recent data suggests that once statistical models are adjusted for covariates, the association was dramatically lessened [22].

The utilisation of pharmacogenomic data is one potential strategy that has the potential to identify the subset of patients most at risk of clarithromycin’s adverse effects.

The systemic drug concentration of clarithromycin is affected by changes in the levels of cytochrome p4503A4 (CYP3A4) and permeability-glycoprotein (P-gp). A study of people with ABCB1 gene polymorphisms associated with low P-gp activity found that they had higher levels of macrolides than expected for a given dose of azithromycin [23]. Additionally, clinical studies also found raised levels of macrolides in patients co-treated with P-gp inhibitors such as omeprazole [24]. Therefore, it was hypothesised that patients with genetic variants associated with low P-gp activity were more likely to experience adverse cardiac events when prescribed a macrolide.

To investigate this further, a genomic observational study was carried out, including patients prescribed either clarithromycin or amoxicillin. In a population of 13,544 individuals, it was identified that clarithromycin prescriptions given to those with lower P-gp activity (based upon two SNPs: rs1045642 and rs1128503-AA genotype) were associated with an increased risk of hospitalisation from a cardiovascular cause at 30 days to 1 year compared to individuals without the AA genotype [25]. Although causality cannot be established from this observational study, it provides a starting point for future studies to determine if pharmacogenomic testing should be implemented into macrolide antibiotic prescribing.

### 4.3. Polygenic Risk Scores

The benefits of individual genetic variants have been the primary focus of pharmacogenomic studies to date. Looking to the future, it is vital to consider the many complex gene-drug interactions that occur simultaneously throughout the body. Often more than one gene contributes to this complex process, and combining these variants can provide prescribers with a clinical tool to help steer their decision-making process. This is demonstrated in the additional analysis of the FOURIER trial (Further Cardiovascular Outcomes Research with PCSK9 Inhibition in Subjects with Elevated Risk), which revealed the benefits of utilising genetic risk scores. In a cohort of over 14,000 patients with atherosclerotic disease, a genetic risk score comprised of 27 loci that had been previously shown to be associated with myocardial infarction was calculated for each participant. The study identified that those with high genetic risk, irrespective of clinical risk, had the greatest risk of cardiovascular events and exhibited the largest benefit from PSCK9 inhibition [26].

Genetic risk scores can also play an important role in reducing the risk of adverse drug effects in cardiovascular therapies. An example of this includes nicorandil, an anti-anginal agent currently recommended as a second-line therapy by the European Society of Cardiology guidelines [27]. Nicorandil’s use is cautioned in diverticular disease due to the increased risk of ulceration and fistula formation. In the western world, it is estimated that over 50% of the population have diverticular disease, with many people exhibiting no symptoms of this [28]. No current screening is offered to individuals prior to commencing nicorandil, which gives rise to the risk of severe adverse drug effects.

A recent case report highlights the severity of symptoms associated with nicorandil intolerance for those who have underlying diverticular disease. The patient developed multiple fistulas between his bladder and rectum, requiring major surgery with subsequent urosepsis admissions, resulting in long term catheter use. The individual remained on nicorandil therapy for 7 years before the connection was made between nicorandil and his symptoms, which resulted in a change of his anti-anginal medication [29].

Pharmacogenomics provides a potential strategy to identify those at increased risk of diverticular disease prior to commencement of anti-anginal medication, without the expense and risk of a colonoscopy. A recent study identified in a genotyped cohort that the genetic risk of diverticular disease predicted early stoppage of nicorandil, which was used as a surrogate marker of nicorandil intolerance [30]. They utilised a previous genome-wide association study (GWAS) which identified SNPs associated with diverticular disease. The GWAS identified 48 variants associated with diverticular disease, 27 of which were replicable in a further European cohort [31]. The nicorandil study found this replicable variant score to be strongly associated with stopping nicorandil therapy early in both univariate and multivariate analyses. This trend was not observed in an isosorbide mononitrate control analysis. This study highlights the potential for a pharmacogenomic based prescribing strategy for anti-anginal therapy, suggesting those of high genetic risk of diverticular disease could be prescribed an alternative agent, such as ranolazine.

## 5. Implementing Pharmacogenomics into Clinical Practice

Despite major advances in the field of pharmacogenomics, much work remains in order to integrate these new prescribing strategies into daily practice. UK general practitioners have raised concerns surrounding the following: cost-effectiveness of the implementation of pharmacogenomics to primary care, how primary care teams will be educated on pharmacogenomic developments and the ethical/legal issues surrounding its use [32]. Reassuringly, across Europe, there is interest and a generally positive attitude among healthcare professionals in favour of pharmacogenomics. The vast majority felt it was relevant to their current practice, despite uncertainties regarding their own knowledge of the subject [33].

Larger hospitals also have many obstacles to overcome prior to the routine implementation of pharmacogenomics into prescribing practice. One project that is currently in progress at our local centre uses pharmacogenomic data to aid prescription choices in individuals who have had an ischaemic stroke [34]. For this project, additional IT systems have had to be generated to link with prescribing systems. This project is based upon a single gene variant and the relevant drug; yet it has required a lot of work to implement a successful system. Once completed, this study will hopefully provide insight and strategy for other centres wishing to implement a precision medicine pathway into their practice. The project has highlighted the potential challenges that are likely to be encountered when adding multiple gene variants and drugs into the equation.

It is yet to be decided which healthcare teams will take ownership of implementing a pharmacogenomic based prescribing system. Currently, secondary care settings undertake most genetic testing and then communicate recommendations to primary care. This is unlikely to be sustainable long-term as the vast majority of prescribing takes place in the community. Studies from the Netherlands have shown that 95% of the population have at least one gene-variant related to the 80 drug-gene interactions identified by the Dutch Pharmacogenetics Working Group (DPWG, Stanford, CA, USA). Twenty-six of these therapies were readily prescribed to large cohorts of people in the community [35]. General Practitioners are increasingly being presented with commercial genetic testing data that their patients have carried out independently. It is therefore crucial that efforts are made to educate and empower primary care services to deal with this new trend. Interestingly, there is much less variation between IT systems implemented across primary care in the UK compared to secondary care. Many large hospitals still have a mix of electronic medical records and paper notes, with little unity across major centres. This is in stark contrast to primary care, where almost all documentation is on electronic systems, and many practices use the same software. Pharmacogenomics, large data and electronic medical records are inherently linked, which provides a further argument that the implementation of pharmacogenomics needs to now focus on the primary care setting.

Despite the many challenges that exist, efforts have been made to implement pharmacogenomics into daily practice and aim to pave the way for others to follow. One potential strategy, implemented at the Mayo Clinic (Rochester, MN, USA), is to have an alert system that fires when a drug is prescribed that is linked to a genetic test. At the Mayo Clinic, there are 17 examples of drug-gene pairs which are highlighted to the prescriber. It is then up to the physician whether they wish to order the corresponding genetic test prompted by the alert. The long-term goal for the clinic is to have pharmacogenomic data pre-emptively available for all patients so that there would be no delay prior to drug commencement [36]. Large scale projects are also underway among multiple sites, including the PREPARE study (PREemptive Pharmacogenomic testing for prevention of Adverse Drug Reactions), which will investigate whether pharmacogenomic based prescribing lowers adverse drug reactions within the first 12 weeks of a new drug treatment. The study aims to recruit around 7000 patients and will consider 44 genetic variants from 12 genes. These genetic variants will guide the prescription choices of 42 drugs with DPWG guidance. Further information regarding cost-effectiveness and clinical utility will hopefully be available after the study has been completed [37]. In the UK, the Chief Medical Officer’s 2016 annual report called for the ‘transformation of patient care through the systemic use of genomics’. The report made recommendations addressing the changes needed in NHS infrastructure and clinical training. These findings are in keeping with the issues identified within this review, and it is reassuring that appropriate investments have been made, such as the UK Life Sciences Sector deal of £65 million, aimed at a collaborative approach for health and data science integration [38].

**Figure 2:**
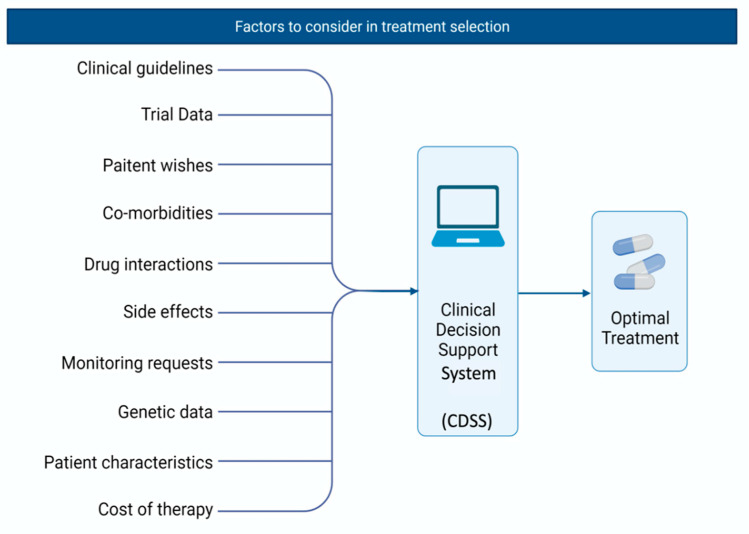
Depiction of an ideal clinical decision support system (CDSS).

Ultimately, the success of pharmacogenomic prescribing will rest on the interface in which healthcare providers interact with the system. A well-designed clinical decision support system (CDSS) that compliments the physician’s normal practice is needed (Figure 2). CDSSs have been a part of current healthcare systems across the world for a number of years and aim to provide clinicians with important information to assist with their patient management decisions. Traditionally, these systems have been ‘knowledge based’, formed around rules related to the literature, pharmacology or patient evidence. An output or action would then be produced based on the evaluation of the set rules. Due to advances in technology, non-knowledge based systems have been created which rely on artificial intelligence to generate their output [39]. Machine learning is an exciting future strategy for the implementation of pharmacogenomic based prescribing due to its complexity and evolving nature. However, additional issues may arise regarding the accountability of prescriptions if the decision-making processes cannot be followed by the prescribers.

## 6. Conclusions

Among healthcare professionals, there is a desire to further the role of pharmacogenomics into clinical practice. Technological advances coupled with educational opportunities across both primary and secondary care will help achieve this. Within this review, the current pharmacogenomic guidelines have been explored in detail, and a selection of exciting future targets for pharmacogenomic based prescribing have been discussed. We have also shown the challenges of integrating pharmacogenomics into everyday practice and highlighted the work taking place to break down these barriers. It is forgivable to temporarily lose sight of the patient when evaluating the abundance of data produced by studies carried out to further the field of pharmacogenomics. One topic that is rarely mentioned is how we translate our genomic knowledge to the patients putting their trust in us. Today, patients are integrally involved in their prescribing choices, which is key to improving medication compliance. Regardless of the future success of pharmacogenomic integration to primary care, genomic-based prescribing will only ever partly influence the treatment choice for our patients. Medicines are not prescribed for genes; the pharmacogenomic advances are an exciting piece of the precision medicine picture that ultimately aims to give the right treatment to the right person at the right time.

## Data Availability

Not applicable.

## References

[1] Al Hamid A., Ghaleb M., Aljadhey H., Aslanpour Z. (2014). A systematic review of hospitalization resulting from medicine-related problems in adult patients. Br. J. Clin. Pharmacol..

[2] Pirmohamed M., James S., Meakin S. (2004). Adverse drug reactions as cause of admission to hospital: Prospective analysis of 18,820 patietns. BMJ.

[3] Rollinson V., Turner R., Pirmohamed M. (2020). Pharmacogenomics for primary care: An overview. Genes.

[4] US Food and Drug Administration (2021). Table of Pharmacogenomic Biomarkers in Drug Labeling. https://www.fda.gov/drugs/science-and-research-drugs/table-pharmacogenomic-biomarkers-drug-labeling.

[5] PharmGKB (2020). Clinical Guideline Annotations. https://www.pharmgkb.org/guidelineAnnotations.

[6] Johnson J.A., Caudle K.E., Gong L., Whirl-Carrillo M., Stein C.M., Scott S.A., Lee M.T., Gage B.F., Kimmel S.E., Perera M.A. (2017). Clinical Pharmacogenetics Implementation Consortium (CPIC) Guideline for Pharmacogenetics-Guided Warfarin Dosing: 2017 Update. Clin. Pharmacol. Ther..

[7] Lee C., Goldstein J., Pieper J. (2002). Cytochrome P450 2C9 polymorphisms: A comprehensive review of the in-vitro and human data. Pharmacogenetics.

[8] Magavern E.F., Kaski J.C., Turner R.M., Janmohamed A., Borry P., Pirmohamed M. (2021). The Interface of Therapeutics and Genomics in Cardiovascular Medicine. Cardiovasc. Drugs Ther..

[9] Mega J., Walker J., Ruff C.T., Vandell A.G., Nordio F., Deenadayalu N., Murphy S.A., Lee J., Mercuri M.F., Giugliano R.P. (2015). Genetics and the clinical response to warfarin and edoxaban: Findings from the randomised, double-blind ENGAGE AF-TIMI 48 trial. Lancet.

[10] Gage B., Bass A., Lin H., Woller S.C., Stevens S.M., Al-Hammadi N., Li J., Rodríguez T., Miller J.P., McMillin G.A. (2017). Effect of Genotype-Guided Warfarin Dosing on Clinical Events and Anticoagulation Control Among Patients Undergoing Hip or Knee Arthroplasty: The GIFT Randomized Clinical Trial. JAMA.

[11] Scott S.A., Sangkuhl K., Stein C.M., Hulot J.S., Mega J.L., Roden D.M., Klein T.E., Sabatine M.S., Johnson J.A., Shuldiner A.R. (2013). Clinical pharmacogenetics implementation consortium guidelines for CYP2C19 genotype and clopidogrel therapy: 2013 update. Clin. Pharmacol. Ther..

[12] Tornio A., Flynn R., Morant S., Velten E., Palmer C.N.A., MacDonald T.M., Doney A.S.F. (2018). Investigating Real-World Clopidogrel Pharmacogenetics in Stroke Using a Bioresource Linked to Electronic Medical Records. Clin. Pharmacol. Ther..

[13] Claassens D.M.F., Vos G.J.A., Bergmeijer T.O., Hermanides R.S., van ’t Hof A.W.J., van der Harst P., Barbato E., Morisco C., Tjon Joe Gin R.M., Asselbergs F.W. (2019). A Genotype-Guided Strategy for Oral P2Y 12 Inhibitors in Primary PCI. N. Engl. J. Med..

[14] Pereira N., Farkouh M., So D., Lennon R., Geller N., Mathew V., Bell M., Bae J.-H., Jeong M.H., Chavez I. (2020). Effect of Genotype-Guided Oral P2Y12 Inhibitor Selection vs Conventional Clopidogrel Therapy on Ischemic Outcomes After Percutaneous Coronary Intervention: The TAILOR-PCI Randomized Clinical Trial. JAMA.

[15] NICE (2010). National Institute for Health and Care Excellence. Clopidogrel and Modified-Release Dipyridamole for the Prevention of Occlusive Vascular Events. NICE Technology Appraisal Guidance.

[16] Ramsey L.B., Johnson S.G., Caudle K.E., Haidar C.E., Voora D., Wilke R.A., Maxwell W.D., McLeod H.L., Krauss R.M., Roden D.M. (2014). The Clinical Pharmacogenetics Implementation Consortium Guideline for SLCO1B1 and Simvastatin-Induced Myopathy: 2014 Update. Clin. Pharmacol. Ther..

[17] Vassy J.L., Michael Gaziano J., Green R.C., Ferguson R.E., Advani S., Miller S.J., Chun S., Hage A.K., Seo S.J., Majahalme N. (2020). Effect of pharmacogenetic testing for statin myopathy risk vs usual care on blood cholesterol a randomized clinical trial. JAMA Netw. Open.

[18] Duan Q.L., Nikpoor B., Dubé M.P., Molinaro G., Meijer I.A., Dion P., Rochefort D., Saint-Onge J., Flury L., Brown N.J. (2005). A variant in XPNPEP2 is associated with angioedema induced by angiotensin I-converting enzyme inhibitors. Am. J. Hum. Genet..

[19] Pare G., Kubo M., Byrd J.B. (2013). Genetic variants associated with angiotensin-converting enzyme inhibitor-associated angioedema. Pharmacogenet. Genom..

[20] Liau Y., Chua I., Kennedy M.A., Maggo S. (2019). Pharmacogenetics of angiotensin-converting enzyme inhibitor-induced angioedema. Clin. Exp. Allergy.

[21] Jespersen C.M. (2006). Randomised placebo controlled multicentre trial to assess short term clarithromycin for patients with stable coronary heart disease: CLARICOR trial. Br. Med. J..

[22] Polgreen L.A., Riedle B.N., Cavanaugh J.E., Girotra S., London B., Schroeder M.C., Polgreen P.M. (2018). Estimated cardiac risk associated with macrolides and fluoroquinolones decreases substantially when adjusting for patient characteristics and comorbidities. J. Am. Heart Assoc..

[23] He X.J., Zhao L.M., Qiu F., Sun Y.X., Li-Ling J. (2009). Influence of ABCB1 gene polymorphisms on the pharmacokinetics of azithromycin among healthy Chinese Han ethnic subjects. Pharmacol. Rep..

[24] Gustavson L.E., Kaiser J.F., Edmonds A.L., Locke C.S., DeBartolo M.L., Schneck D.W. (1995). Effect of omeprazole on concentrations of clarithromycin in plasma and gastric tissue at steady state. Antimicrob. Agents Chemother..

[25] Mordi I.R., Chan B.K., Yanez N.D., Palmer C.N.A., Lang C.C., Chalmers J.D. (2020). Genetic and pharmacological relationship between P-glycoprotein and increased cardiovascular risk associated with clarithromycin prescription: An epidemiological and genomic population-based cohort study in Scotland, UK. PLoS Med..

[26] Marston N.A., Kamanu F.K., Nordio F., Gurmu Y., Roselli C., Sever P.S., Pedersen T.R., Keech A.C., Wang H., Pineda A.L. (2020). Predicting Benefit from Evolocumab Therapy in Patients with Atherosclerotic Disease Using a Genetic Risk Score. Circulation.

[27] Knuuti J., Wijns W., Saraste A., Capodanno D., Barbato E., Funck-Brentano C., Prescott E., Storey R.F., Deaton C., Cuisset T. (2019). 2019 ESC Guidelines for the diagnosis and management of chronic coronary syndromes. Eur. Heart J..

[28] Weizman A.V., Nguyen G.C. (2011). Diverticular disease: Epidemiology and management. Can. J. Gastroenterol..

[29] Noyes J.D., Mordi I.R., Zeb Q., Lang C.C. (2021). Nicorandil-induced colovesical fistula in a patient with diverticular disease. Clin. Case Rep..

[30] Noyes J.D., Mordi I.R., Doney A.S., Palmer C.N.A., Pearson E.R., Lang C.C. (2020). Genetic Risk of Diverticular Disease Predicts Early Stoppage of Nicorandil. Clin. Pharmacol. Ther..

[31] Schafmayer C., Harrison J.W., Buch S., Lange C., Reichert M.C., Hofer P., Cossais F., Kupcinskas J., von Schönfels W., Schniewind B. (2019). Genome-wide association analysis of diverticular disease points towards neuromuscular, connective tissue and epithelial pathomechanisms. Gut.

[32] Rafi I., Crinson I., Dawes M., Rafi D., Pirmohamed M., Walter F.M. (2020). The implementation of pharmacogenomics into UK general practice: A qualitative study exploring barriers, challenges and opportunities. J. Community Genet..

[33] Just K.S., Steffens M., Swen J.J., Patrinos G.P., Guchelaar H.J., Stingl J.C. (2017). Medical education in pharmacogenomics—results from a survey on pharmacogenetic knowledge in healthcare professionals within the European pharmacogenomics clinical implementation project Ubiquitous Pharmacogenomics (U-PGx). Eur. J. Clin. Pharmacol..

[34] Doney A.S., Jefferson E. (2021). Learning Healthcare System for Stroke Precision Medicine Pathway (P4Me). Population Health and Genomics.

[35] Rigter T., Jansen M.E., de Groot J.M., Janssen S.W.J., Rodenburg W., Cornel M.C. (2020). Implementation of Pharmacogenetics in Primary Care: A Multi-Stakeholder Perspective. Front. Genet..

[36] Weinshilboum R.M., Wang L. (2017). Pharmacogenomics: Precision Medicine and Drug Response. Mayo Clin. Proc..

[37] Van Der Wouden C.H., Cambon-Thomsen A., Cecchin E., Cheung K.C. (2017). Implementing Pharmacogenomics in Europe: Design and Implementation Strategy of the Ubiquitous Pharmacogenomics Consortium. Clin. Pharmacol. Ther..

[38] Stark Z., Dolman L., Manolio T.A., Ozenberger B., Hill S.L., Caulfied M.J., Levy Y., Glazer D., Wilson J., Lawler M. (2019). Integrating Genomics into Healthcare: A Global Responsibility. Am. J. Hum. Genet..

[39] Sutton R.T., Pincock D., Baumgart D.C., Sadowski D.C., Fedorak R.N., Kroeker K.I. (2020). An overview of clinical decision support systems: Benefits, risks, and strategies for success. NPJ Digit. Med..

